# Machine learning application for prediction of locoregional recurrences in early oral tongue cancer: a Web-based prognostic tool

**DOI:** 10.1007/s00428-019-02642-5

**Published:** 2019-08-17

**Authors:** Rasheed Omobolaji Alabi, Mohammed Elmusrati, Iris Sawazaki-Calone, Luiz Paulo Kowalski, Caj Haglund, Ricardo D. Coletta, Antti A. Mäkitie, Tuula Salo, Ilmo Leivo, Alhadi Almangush

**Affiliations:** 1 Department of Industrial Digitalization, School of Technology and Innovations, University of Vaasa, Vaasa, Finland; 2 Oral Pathology and Oral Medicine, Dentistry School, Western Parana State University, Cascavel, PR Brazil; 3 Department of Head and Neck Surgery and Otorhinolaryngology, A.C. Camargo Cancer Center, São Paulo, SP Brazil; 4 Research Programs Unit, Translational Cancer Biology, University of Helsinki, Helsinki, Finland; 5 Department of Surgery, University of Helsinki and Helsinki University Hospital, Helsinki, Finland; 6 Department of Oral Diagnosis, School of Dentistry, University of Campinas, Piracicaba, São Paulo, Brazil; 7 Department of Otorhinolaryngology – Head and Neck Surgery, University of Helsinki and Helsinki University Hospital, Helsinki, Finland; 8 Research Programme in Systems Oncology, Faculty of Medicine, University of Helsinki, Helsinki, Finland; 9 Division of Ear, Nose and Throat Diseases, Department of Clinical Sciences, Intervention and Technology, Karolinska Institutet and Karolinska University Hospital, Stockholm, Sweden; 10 Department of Pathology, University of Helsinki, Helsinki, Finland; 11 Department of Oral and Maxillofacial Diseases, University of Helsinki, Helsinki, Finland; 12 Cancer and Translational Medicine Research Unit, Medical Research Center Oulu, University of Oulu and Oulu University Hospital, Oulu, Finland; 13 Institute of Biomedicine, Pathology, University of Turku, Turku, Finland; 14 Faculty of Dentistry, University of Misurata, Misurata, Libya

**Keywords:** Oral tongue cancer, Artificial neural network, Machine learning, Locoregional recurrence, Prediction

## Abstract

Estimation of risk of recurrence in early-stage oral tongue squamous cell carcinoma (OTSCC) remains a challenge in the field of head and neck oncology. We examined the use of artificial neural networks (ANNs) to predict recurrences in early-stage OTSCC. A Web-based tool available for public use was also developed. A feedforward neural network was trained for prediction of locoregional recurrences in early OTSCC. The trained network was used to evaluate several prognostic parameters (age, gender, T stage, WHO histologic grade, depth of invasion, tumor budding, worst pattern of invasion, perineural invasion, and lymphocytic host response). Our neural network model identified tumor budding and depth of invasion as the most important prognosticators to predict locoregional recurrence. The accuracy of the neural network was 92.7%, which was higher than that of the logistic regression model (86.5%). Our online tool provided 88.2% accuracy, 71.2% sensitivity, and 98.9% specificity. In conclusion, ANN seems to offer a unique decision-making support predicting recurrences and thus adding value for the management of early OTSCC. To the best of our knowledge, this is the first study that applied ANN for prediction of recurrence in early OTSCC and provided a Web-based tool.

**Electronic supplementary material:**

The online version of this article (10.1007/s00428-019-02642-5) contains supplementary material, which is available to authorized users.

## Introduction

Oral tongue squamous cell carcinoma (OTSCC) typically displays aggressive behavior even at an early stage [1, 2]. Inaccurate assessment of OTSCC behavior may lead to improper management either as ineffective treatment or as unnecessary overtreatment. Therefore, identifying patients with low-risk or high-risk OTSCC can influence management decision-making and guide the selection of treatment approach. Several prognostic markers have been suggested to improve the prognostication of OTSCC [3, 4]. The advantages of evaluating some histopathologic prognostic markers in the examination of routine hematoxylin and eosin (HE)-stained slides include their low cost and time-saving aspects (as there is no need for additional staining) as well as the fact that these markers are potentially ready to be included in the routine pathology reports. Such advantages have motivated researchers to study various histopathologic features, and the recent evidence has confirmed the prognostic value of certain markers including, for example, tumor budding [5], depth of invasion [6], worst pattern of invasion [7], and perineural invasion [8]. It is necessary to mention that the previous studies on these markers have used traditional tools for data analysis, which have not produced any simple approach to utilize them as multiple prognostic factors should be applied to aid decision making.

**Fig. 1:**
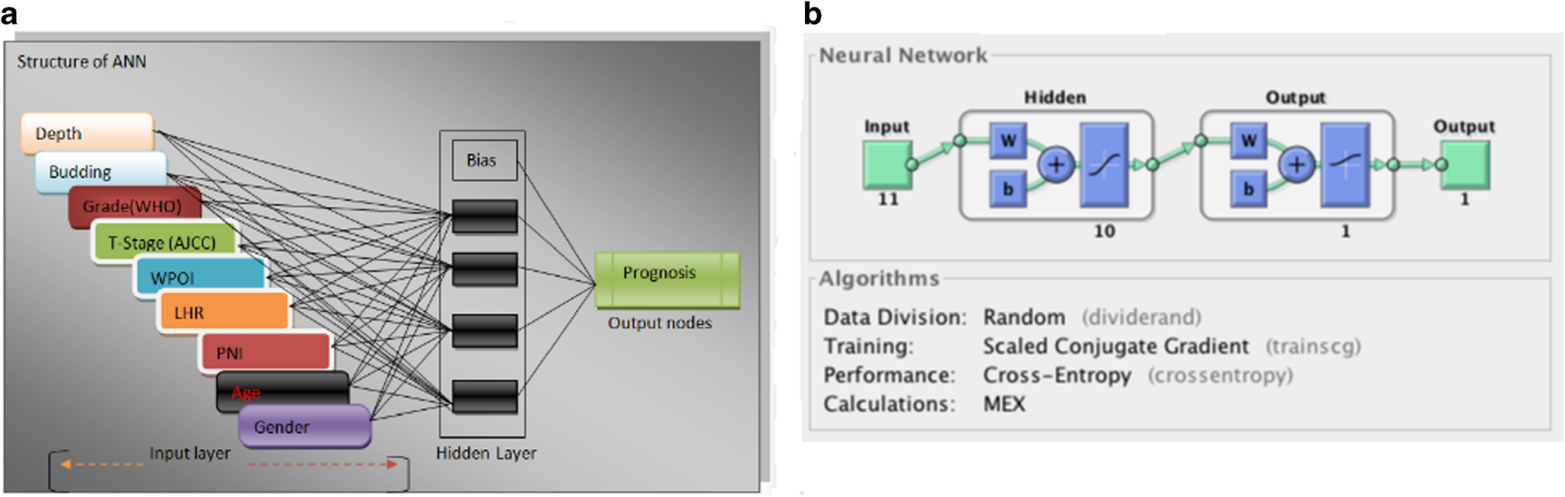
Structure of ANN with prognostic factors for training the network (WPOI worst pattern of invasion, LHR lymphocytic host response, PNI perineural invasion)

The use of machine learning, a branch of artificial intelligence, in medical applications has increased widely in recent years; this has been driven by the rapidly accumulating volume of medical data. Similarly, artificial neural networks (ANNs) are an integral part and a subfield of machine learning. An ANN is an innovative hardware/software model that functions in a way inspired by the human brain [9–12]. In addition, ANN seems effective since the complex relationship between input and output can be accurately modeled with a relatively simple computer programming code. Structurally, ANN comprises input, hidden, and output layers (Fig. 1).

ANNs have an effective learning ability, and they can learn the relationship within a dataset. This effective learning characteristic has made ANN a good choice for predictive inferences that can be used to provide support for clinical decision-making. Many recent studies have applied ANNs for the prognostication of different cancers [9–12]. ANNs are adapted statistical models that analyze data for the prediction of outcomes in medical applications [13, 14], such as in colorectal cancer [15] and acute pancreatitis [16]. Spelt and collaborators applied ANN to predict survival in colorectal cancer, revealing that ANN produces better C-index than Cox regression [17].

The use of ANNs specifically for early OTSCC has not been previously studied. Thus, this study examined the use of ANN in prognostication of early OTSCC. We examined the use of ANNs to estimate the risk of locoregional recurrence in early-stage OTSCC. The neural network toolbox of MATLAB (R2018b version) was used to create, train, and simulate ANN for pattern recognition and classification [18]. Furthermore, the Microsoft Azure machine learning studio (Azure, 2018) was used to develop a Web-based prognostic estimator that can provide a prediction for each individual case in daily practice.

## Material and methods

### Patients

The clinicopathologic characteristics of 311 patients with cT1-2cN0cM0 OTSCC treated between 1979 and 2009 at the University Hospitals of Helsinki, Oulu, Turku, Tampere, and Kuopio (all in Finland) and at the A.C. Camargo Cancer Center in Sao Paulo, Brazil, were collected. The histopathologic parameters are briefly summarized in Table 1. The use of patient samples and data inquiry in this study were approved by the Finnish National Supervisory Authority for Welfare and Health (VALVIRA), and by the Ethics Committee in Research of the Piracicaba Dental School, University of Campinas, São Paulo, Brazil.

**Table 1:** Summary of histopathologic parameters included for neural network analysis and development of the Web-based tool

Variable	Categories	Definition	Total	Recurrence
WHO grade				
	Grade I	Well-differentiated tumor	105	28
	Grade II	Moderately differentiated tumor	131	38
	Grade III	Poorly differentiated tumor	75	23
Tumor budding*				
	None	There is no tumor budding	114	26
	Low	Tumor has less than five buds	102	24
	High	Tumor has five buds or more at the invasive front	95	39
Depth of invasion				
	Superficial	Tumor less than 4 mm in depth	116	26
	Deep	Tumor with 4 mm in depth or deeper	195	63
Worst pattern of invasion (WPOI)				
	Type 1; Type 2; Type 3**	Pushing border; finger-like growth; large tumor islands	78	15
	Type 4	Small tumor islands (≤ 15 cancer cells)	190	61
	Type 5	Tumor satellites	43	13
Lymphocytic host response (LHR)				
	Type 1	Strong	53	16
	Type 2	Intermediate	116	35
	Type 3	Weak	142	38
Perineural invasion (PNI)				
	Absent	PNI was not observed	269	73
	Present	PNI was observed	42	16

*Tumor budding defined as a single cancer cell or cancer cluster of four cancer cells or less

**Types 1, 2, and 3 of worst pattern of invasion were considered in one risk group

### Prognostic parameters

Clinicopathologic variables including age, gender, T stage (AJCC 7th), and WHO grade were included as classic prognostic factors. All histopathologic parameters were evaluated on postoperative surgical specimens stained with routine hematoxylin and eosin. The histopathologic parameters include the WHO histological grade, tumor budding, depth of invasion, worst pattern of invasion (WPOI), lymphocytic host response (LHR), and perineural invasion (PNI). We selected these prognostic factors based on our recent reports on the significance of tumor budding [19, 20], depth of invasion, and worst pattern of invasion in early OTSCC [7]. Of note, a recent study on a large cohort of OSCC [21] underlined the prognostic significance of all the prognostic factors that we used to construct the ANN.

### ANN for prediction of locoregional recurrence

The dataset of 311 cases was loaded into the MATLAB workspace (The MathWorks, Inc., USA). An example of a feedforward neural network used was the basic feedforward network also known as multi-layer perceptron (MLP), with sigmoid hidden and softmax output activation function [22]. It is a two-layer network where the training of the network is based on the definition of a suitable error function, which is optimized with respect to the weights and biases in the network [22].

### Prediction of locoregional recurrence

A supervised learning method was used in this study. After loading the dataset into the MATLAB workspace, prognostic factors including age, gender, stage, WHO histologic grade, tumor budding, tumor depth, WPOI, LHR, and PNI were set as inputs for the neural network, and locoregional recurrence was considered as the output. The neural network representation of the inputs, hidden neurons, and the outputs of the training process is shown in Fig. 1.

**Fig. 2:**
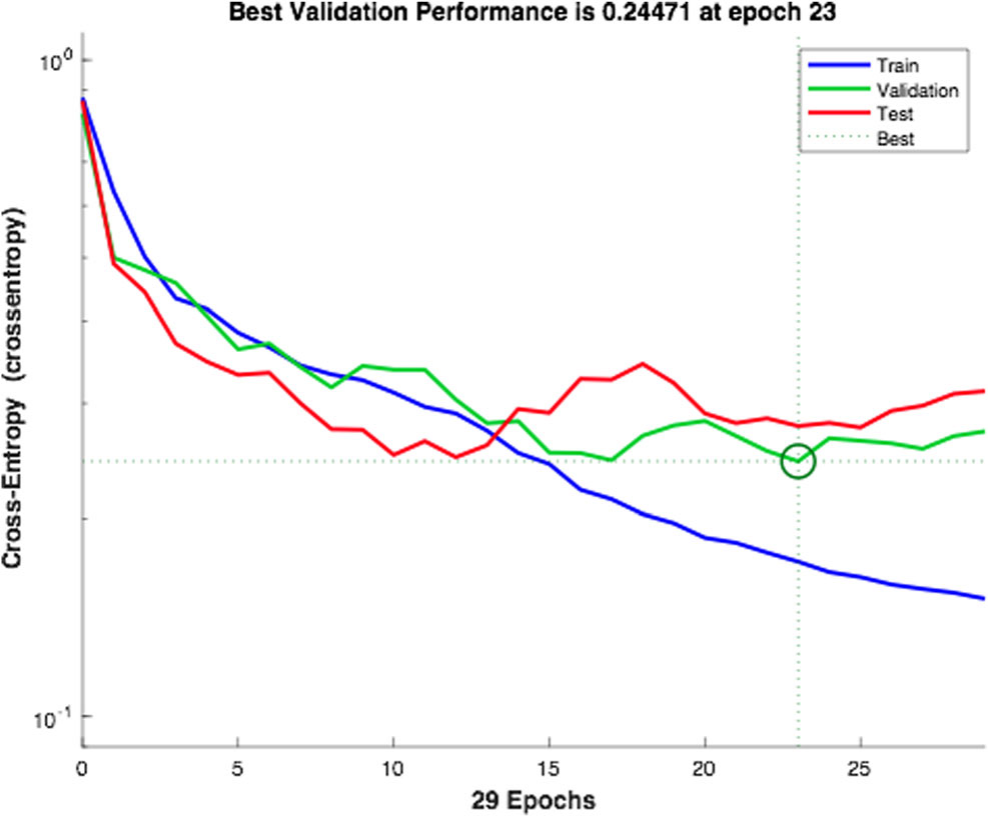
The network training performance measure using cross-entropy

The dataset is usually divided into 70% training, 15% validation, and 15% testing sets [18, 23, 24]. In some instances, the validation and testing sets can be combined and considered to be testing sets only. This was the case with the Azure machine learning studio [25]. The prediction of locoregional recurrence was thought to be a classification task which is a form of pattern recognition. Therefore, the network was trained using *patternnet* function. It creates a standard solution neural network that classifies inputs into a target. The final process involves training the configured network for prediction [26, 27]. Follow-up time and disease-free time were included in the training of the network. The network was trained using scaled conjugate gradient backpropagation and the performance of the network was computed using cross-entropy as shown in Fig. 2. The overall performance of this trained network was measured in terms of accuracy and area under receiving characteristic curve. Additionally, we compared the performance of this ANN model with logistic regression model in terms of accuracy.

### Analyses of the importance of prognostic parameters

To examine the importance of each of the prognostic factors, each factor was removed from the inputs and the network was re-trained. The performance error was observed. This process was repeated for all the inputs shown in Fig. 1. Furthermore, it is important to gain insight into the input variables, recognize the pattern between them, and their level of correlation. Therefore, clustering offers one unique approach to achieve these. It is another excellent application of neural network, though mostly used in unsupervised learning. In this study, clustering was performed with a self-organizing map (SOM). The SOM is the most commonly used type of neural network for clustering. It has a competitive layer with neurons arranged in a grid form and hexagonal topology. The SOM network is trained with the input variables with each of them being connected to each of the neurons using the weight vector. The input data have been visualized in 2D using heatmap. Heatmaps visualize data through setting variations in coloring. The heatmap (weight planes/component planes) showing different input variables is shown in Supplementary Fig. 1. Additionally, the clustering of patients into two groups of either high- or low-risk recurrence is given in Supplementary Fig. 2.

### Implementation of the Web-based prognostic tool

The process of Web deployment using the Azure machine learning Web application templates (Microsoft Corporation, USA) involves two phases. The first phase is to develop a predictive model using a machine learning studio. In the second phase, the predictive model was then accessed and thus set up as a Web service directly from Azure machine learning studio. The Web tool for recurrence prediction can be freely accessed on the Microsoft Azure cloud service [25]. Users of this Web site can enter prognostic factors to generate a personalized estimation of locoregional recurrence for the patient. The Web page is https://predictrecurrence.azurewebsites.net/Default.aspx. We tested the accuracy of our Web-based prognostic tool using 59 new cases of early OSCC treated between 1998 to 2008 at the UOPECCAN Cancer Hospital (Cascavel, Parana, Brazil). These cases were included in our previous study [28], but they were not included in the training of the ANN and were not included in the development of our Web page.

## Results

The clinicopathologic characteristics of these patients have been previously reported [19]. This cohort consists of 165 men and 146 women. The distribution of tumors according to their diameter showed that 124 cases were staged T1 and 187 were T2. The number of patients with disease recurrences was 89 (28.6%). All cases were clinically N0 and M0. Similarly, the new cohort of 59 cases (46 men, 13 women) differs from the first one of 311 cases used in the training. The distribution according to tumor diameter showed that 22 patients had stage T1 and 37 stage T2. In terms of the distribution according to tumor budding, 14 patients showed no budding, 19 patients had less than five buds, and 26 patients had five buds or more. The mean age at diagnosis within this cohort was 56.2 (range 31–84). The number of patients with a disease recurrence was 19 (32.2%) in this cohort that was used to test the Web-based tool.

**Fig. 3:**
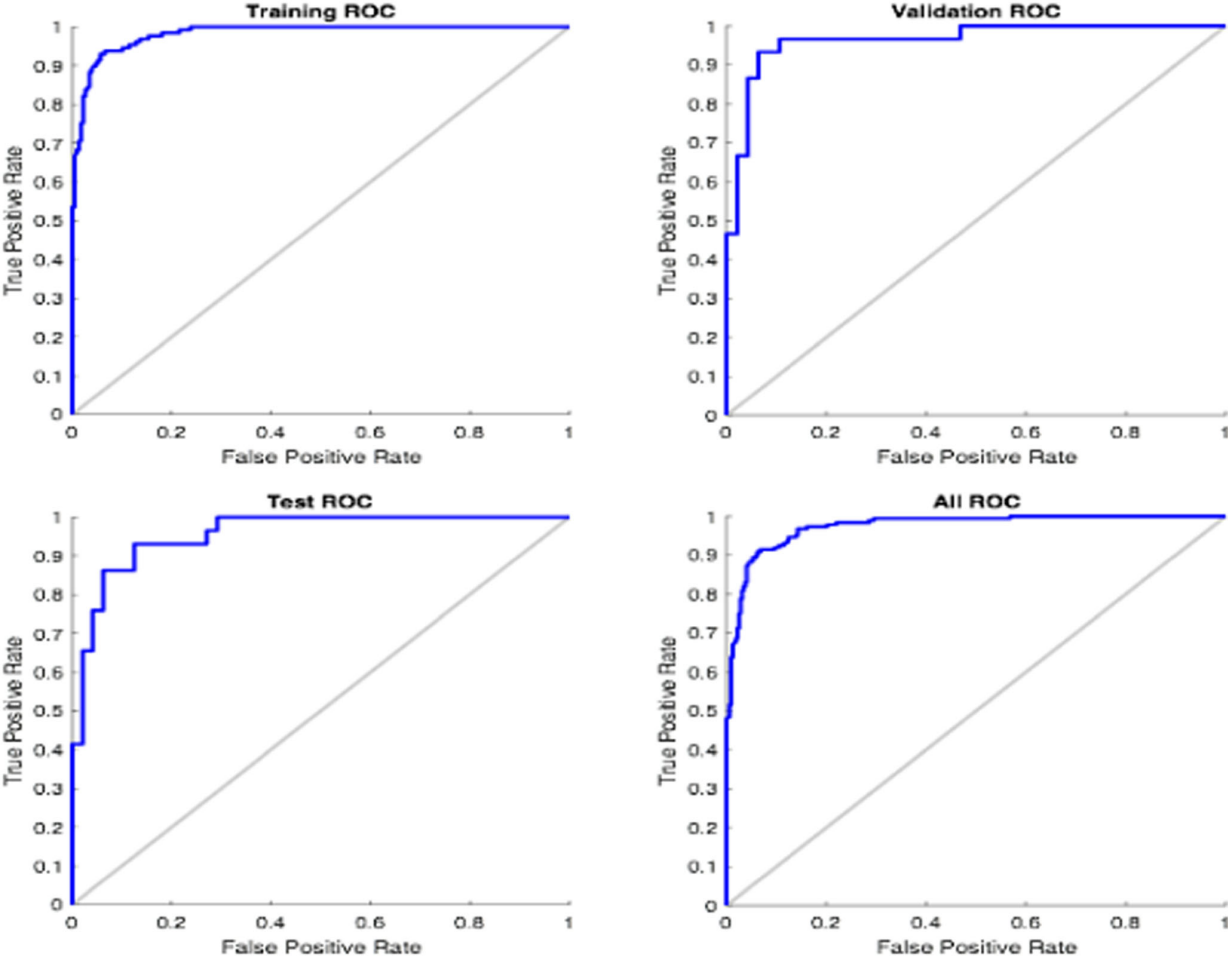
The ROC curve of the trained network in MATLAB

**Fig. 4:**
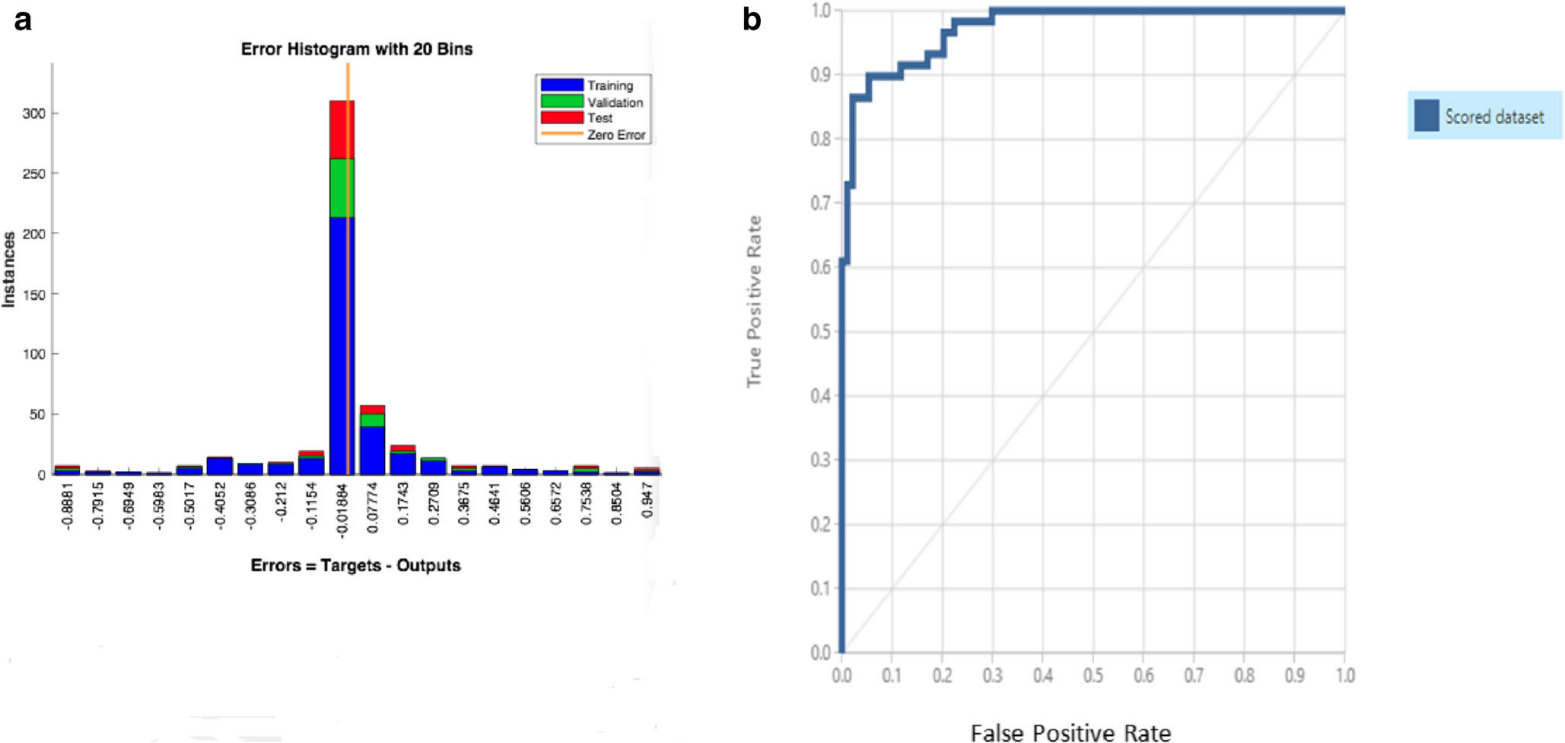
**a** Error histogram showing the difference between the targets and outputs. **b** An indicative receiver operating characteristics (ROC) curve from Azure for the Web deployment

Our ANN model recognized tumor budding and depth of invasion as the most important histopathological prognostic parameters for the network to effectively predict locoregional recurrence. The heatmap presented (Supplementary Fig. 1) showed that the prognostic significance of input variables were independent. Also, the SOM network appeared to have clustered the patient into two distinct groups of high- and low-risk recurrence (Supplementary Fig. 2). In terms of accuracy of the network, the ANN yielded an overall accuracy of 92.7%. The accuracy of the ANN was higher than that given by the logistic regression model which gave an accuracy of 86.5%. The receiving operating characteristic curve of the network is given in Fig. 3. The error histogram of the training, validation, and testing phases is shown in Fig. 4a.

An overall accuracy of 88.2% was obtained with the Web prognostication tool. This was actually the overall proportion of properly classified instances between the outputs and the targets. Other metrics from the evaluation model included recall, precision, and area under receiving operating characteristics curve (AUC). Recall, which is also known as sensitivity, was 71.2% and specificity was 98.9%. The positive and negative predictive values were 97.7% and 84.5%, respectively. The C-statistics (C-index) value was 97.3%. It is necessary to mention that C-index equals to the area under the receiving operating characteristics (ROC) curve shown in Fig. 4b. The performance measures for both MATLAB and Azure Web services are summarized in Table 2.

**Table 2:** The overall performance measures of the network

Software	Performance measures for the training of the network				
MATLAB	0.24471 Network performance error		92.7% Accuracy		
Azure Machine Learning (ML) Studio	88.2% Accuracy	71.2% Sensitivity	98.9% Specificity	0.824 F1 Score	97.3% C-index/AUC
	97.7% Positive predictive value (PPV)		84.5% Negative predictive value (NPV)		

### Testing/validation of the Web site with new cases

The Web site was tested with a new cohort of cases. Of the 59 cases tested, 48 cases were predicted correctly while 11 cases gave incorrect predictions when compared with the actual status of locoregional recurrence recorded by the hospital. For this new cohort of cases, an 81.4% overall accuracy was achieved using this Web-based tool. A sensitivity value of 78.9% was recorded indicating more than two thirds of the cases under consideration. With this high value of sensitivity, false-negative cases would be greatly reduced and would lead to reduction in classification (prediction) error. The overall performance metrics of the tested cohorts using our Web-based prognostic tool is presented in Table 3.

**Table 3:** The performance of the Web-based tool on the newly tested cases

	Patients with OTSCC			
Web-based tool for the prediction of OTSCC recurrences	High-risk OTSCC recurrences	Low-risk OTSCC recurrences	Total	Other performance metrics
	15 True positive	7 False positive	22 Total_Test-positive	68.2% Positive predictive value (PPV)
	4 False negative	33 True negative	37 Total_Test-negative	89.2% Negative predictive value (NPV)
	19 Total_High-risk OTSCC recurrences	40 Total_Low-risk OTSCC recurrences	59 Total_Test-cases	4.5/0.35 Positive/negative likelihood ratios
	78.9% Sensitivity	82.5% Specificity		

Furthermore, a specificity value of 82.5% was achieved with these test cases. In other words, the data from 33/40 patients with “low-risk” truly gave a prediction of “low-risk” in the Web-based tool. A positive predictive value of 68.2% was observed, pointing out the likelihood of a high-risk test result in individuals who actually developed a recurrence. Conversely, a negative predictive value of 89.2% was observed. The latter value indicates the probability of a low-risk result in the Web-based tool in individuals who are cases of a true low risk for recurrence. Finally, from the abovementioned performance information, a positive likelihood ratio (LR^+^) of 4.5 and a negative likelihood ratio (LR^−^) of 0.25 were computed. A LR^+^ indicates how much more likely it is for the Web-based tool to predict a high risk for recurrence compared to a low risk for recurrence. Similarly, the LR^−^ value indicates how much less likely it is for the Web-based tool to predict a low risk for recurrence.

## Discussion

In this study, we explored the use of ANN to predict locoregional recurrences in early-stage oral tongue cancer and we reported a better performance for the ANN compared with logistic regression. We also examined the odd ratios of each of these prognostic parameters. In addition, we developed a Web-based tool that provides the prediction as “low-risk” or “high-risk” of recurrence. The histopathologic parameters used in this study (and involved in our Web-based tool) were selected based on findings in our previous study [19] and our recent meta-analysis of many studies reporting the importance of tumor budding [20]. Depth of invasion and worst pattern of invasion have also shown promising prognostic significance in recent research by our group [7] and others [29, 30]. Perineural invasion was a valuable marker in other recent studies [8, 31]. In early OSCC, Arora et al. [21] have recently introduced a prognostic model including all the histopathologic parameters that were included in our current study. Of note, all histopathologic parameters included in this study can be evaluated using routine hematoxylin and eosin staining, and some of those parameters are routinely included in pathology reports. Moreover, multivariate analyses of many studies have underlined the prognostic significance of the selected parameters [8, 19, 21, 29, 31].

This is the first study that used an ANN and provided a Web tool for the prediction of recurrence in early-stage OTSCC. A neural network seems to have the potential to offer a better approach to data analyses and pattern recognition within data. It can build a nonlinear statistical model to examine biological systems. There is no need to identify key prognostic markers or to form a hypothesis in analyses using ANN. Interestingly, the input variables were shown to be independent of each other (Supplementary Fig. 1) as the connection patterns of these inputs are dissimilar; hence, each of these variables represents a different concept on the target variable. Thus, the issue of collinearity in machine learning is prevented (no two input variables have the same effects on the target variable). Therefore, a band of dark segments from the lower-right region to the upper-right region demonstrate the potential group associated with recurrence of OTSCC (Supplementary Fig. 2).

While age of patient, tumor budding, depth, worst pattern of invasion, and perineural invasion showed significant association in terms of odd ratios to the recurrence of OTSCC, other parameters such as gender, clinical stage, histopathological grade, lymphocytic host response, and follow-up time showed low odd ratios but were included in the neural network as confounders to report independence of the significant markers and to improve the performance of ANN. Our study assessed histopathologic parameters based on postoperative surgical specimens. Therefore, patients that were recognized as “high-risk cases” (according to our Web-based tool) might benefit from postoperative adjuvant treatment (e.g., radiotherapy). Of note, recent research has showed that some histopathologic parameters (e.g., PNI, depth of invasion, and tumor budding) that were included in our study can be evaluated preoperatively either using magnetic resonance imaging [32] or satisfactory diagnostic biopsies [20]. All these are additional parameters to tumor grade, which is routinely reported for preoperative biopsies. Thus, further research should consider examining our Web-based tool in a large cohort with preoperative assessment of these histopathologic parameters. Such approach has the potential of being of great importance for treatment planning.

In the Microsoft Azure machine learning studio, a two-class neural network algorithm was used to develop the Web-based prognostic tool. It was able to produce reasonably well true positive and false negative values in recurrence prediction and had a high precision value of 97.7%. This value is also known as the positive predictive value, which explains the performance of our Web-based tool. The true positive and false positive rates can be inspected in the receiving operating characteristics (ROC) plot, also known as a precision/recall plot, and the corresponding area under the ROC curve (Fig. 4b). In our study, the area under the characteristic curve was 97.3% with a curve that tends towards the upper left corner (Fig. 4b) and far from the diagonal. This suggests a good performance of the model. The values of the likelihood ratios (LR^+^ 4.5/LR^−^ 0.25) implied that our Web-based tool could effectively predict the cases associated with or without a recurrence of OTSCC.

This study also showed that the performance accuracy of ANN was higher than the logistic regression model. Other studies have compared ANN with traditional statistical models. For example, the study by Faradmal et al. demonstrated that the ability of prediction with ANN was higher than with the log-logistic regression model in predicting breast cancer relapse [33]. Similarly, Kazemnejad et al. compared ANN with binary logistic regression based on their performance in differentiating between disease-free patients and patients with impaired glucose tolerance or diabetes mellitus diagnosed by fasting plasma glucose [34].

In this study, the feedforward neural network produced a better performance and predicted the recurrences reasonably well by using enough neurons in the hidden layer. Computing the performance of the network using cross-entropy ensures a trained network that heavily penalizes outputs that are extremely inaccurate, with only little penalty for fairly correct classifications. Thereby, it proved to be a network with good classification capabilities. Hence, our findings indicated that ANN is an effective approach for predicting recurrences in early OTSCC. The Web-based tool provides the prediction as “low-risk” or “high-risk” of recurrence. Thus, the decision of multimodality treatment can be taken for those cases at high risk although they are diagnosed at early stage.

It is important to mention that our Web-based tool was trained with a limited number of cases. Therefore, it is possible that it will miss some predictions. In addition, the values within the follow-up time in months and disease-free time columns are not sufficiently diverse. This means that prediction from the Web-based tool for extremely high values of follow-up time could not be relied upon. Accordingly, feedback from users of this Web-based tool would be greatly appreciated. It is also hoped that this tool would be re-trained at certain intervals for better prediction based on the anticipated feedback for better prediction capacity. In addition, our current neural network did not include some parameters such as margin status and pTNM stage due to unavailability of such information for several cases in our multicenter cohort of six institutions. Thus, we were not able to include these two parameters during the construction of our neural network, and we advise including such parameters in the further development of the neural network of early OTSCC.

In conclusion, the use of ANN is an efficient means to predict recurrence in early OTSCC. The combination of markers that were presented in our Web-based tool were able to predict recurrence successfully. With our Web-based tool, patients could be identified as high or low-risk individuals, which makes it easier to assess their prognoses. Those high-risk cases were identified with aggressive histopathologic characteristics (e.g., high intensity of tumor budding and deep invasion). Thus, such cases might benefit from elective neck dissection and postoperative oncological therapy in addition to an individualized enhanced posttreatment follow-up program. To further develop this Web-based tool, a multicenter setting should be applied to add more data to improve its effectiveness.

## Electronic supplementary material

Supplementary Figure 1. The heatmap of the input variables. (Input 1 = Age, , Input 2 = Gender, Input 3 = Stage, Input 4 = Grade, Input 5 = Tumor Budding, Input 6 = Depth, Input 7 =Worst Pattern of Invasion, Input 8 = Lymphocytic Host Response, Input 9 = Perineural Invasion, Input 10 = Disease free months, Input 11= Follow-up time) (GIF 119 kb)

High resolution image (TIF 122 kb)

Supplementary Figure 2. The U-Matrix (weight distance matrix) showing the SOM neighbor weight distances. (GIF 136 kb)

High resolution image (TIF 109 kb)
