# Supplementary figures and images for: Machine learning application for prediction of locoregional recurrences in early oral tongue cancer: a Web-based prognostic tool
Source: Virchows Arch. 2019 Aug 17;475(4):489–97. doi: 10.1007/s00428-019-02642-5 (PMC6828835; doi:10.1007/s00428-019-02642-5)

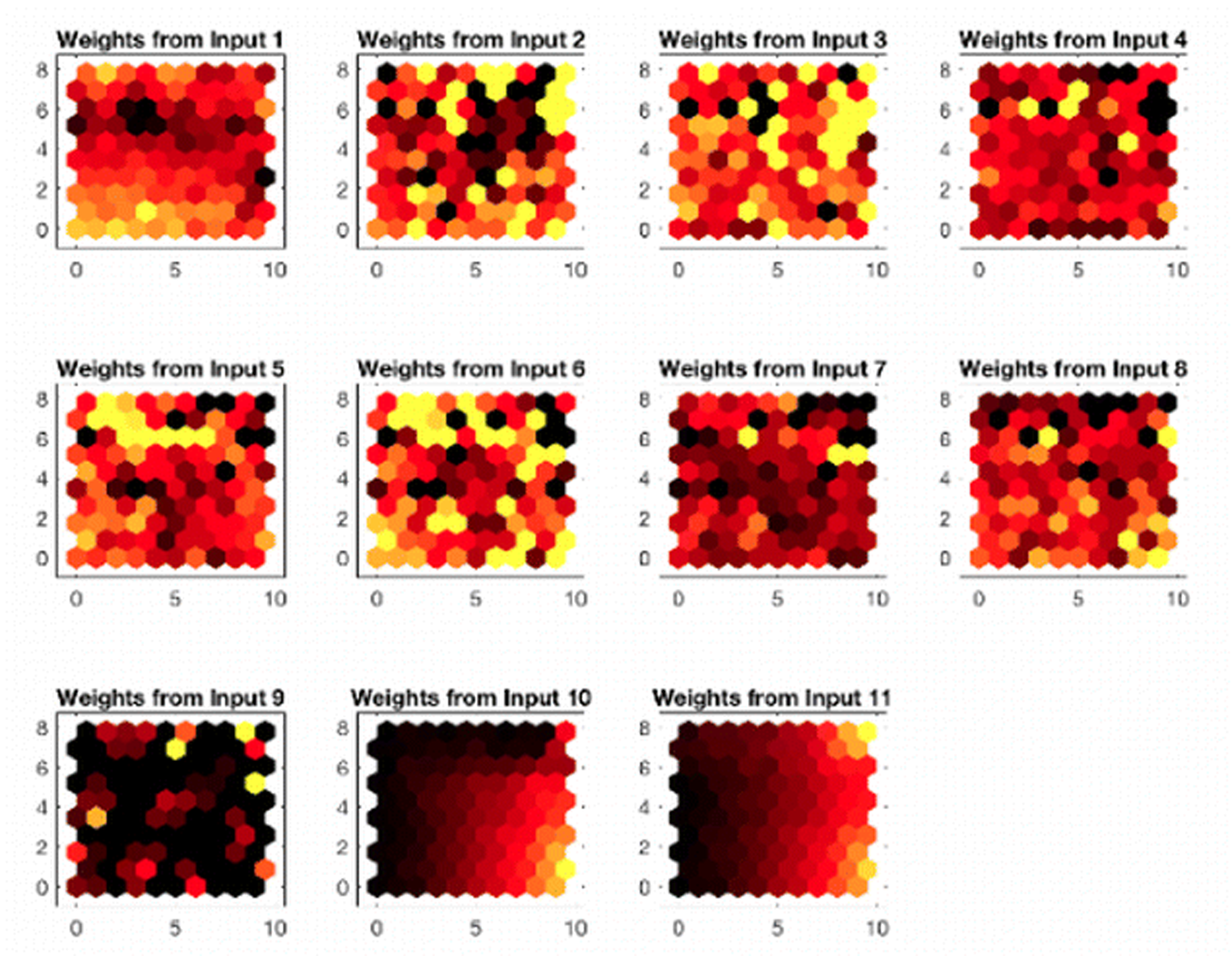

Supplement: Supplementary file 1 — The heatmap of the input variables. (Input 1 = Age, , Input 2 = Gender, Input 3 = Stage, Input 4 = Grade, Input 5 = Tumor Budding, Input 6 = Depth, Input 7 =Worst Pattern of Invasion, Input 8 = Lymphocytic Host Response, Input 9 = Perineural Invasion, Input 10 = Disease free months, Input 11= Follow-up time) (GIF 119 kb) [file 428_2019_2642_Fig5_ESM.png]

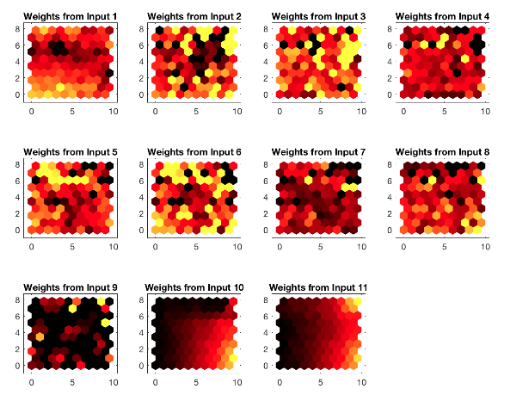

Supplement: Supplementary file 2 — High resolution image (TIF 122 kb) [file 428_2019_2642_MOESM1_ESM.tif]

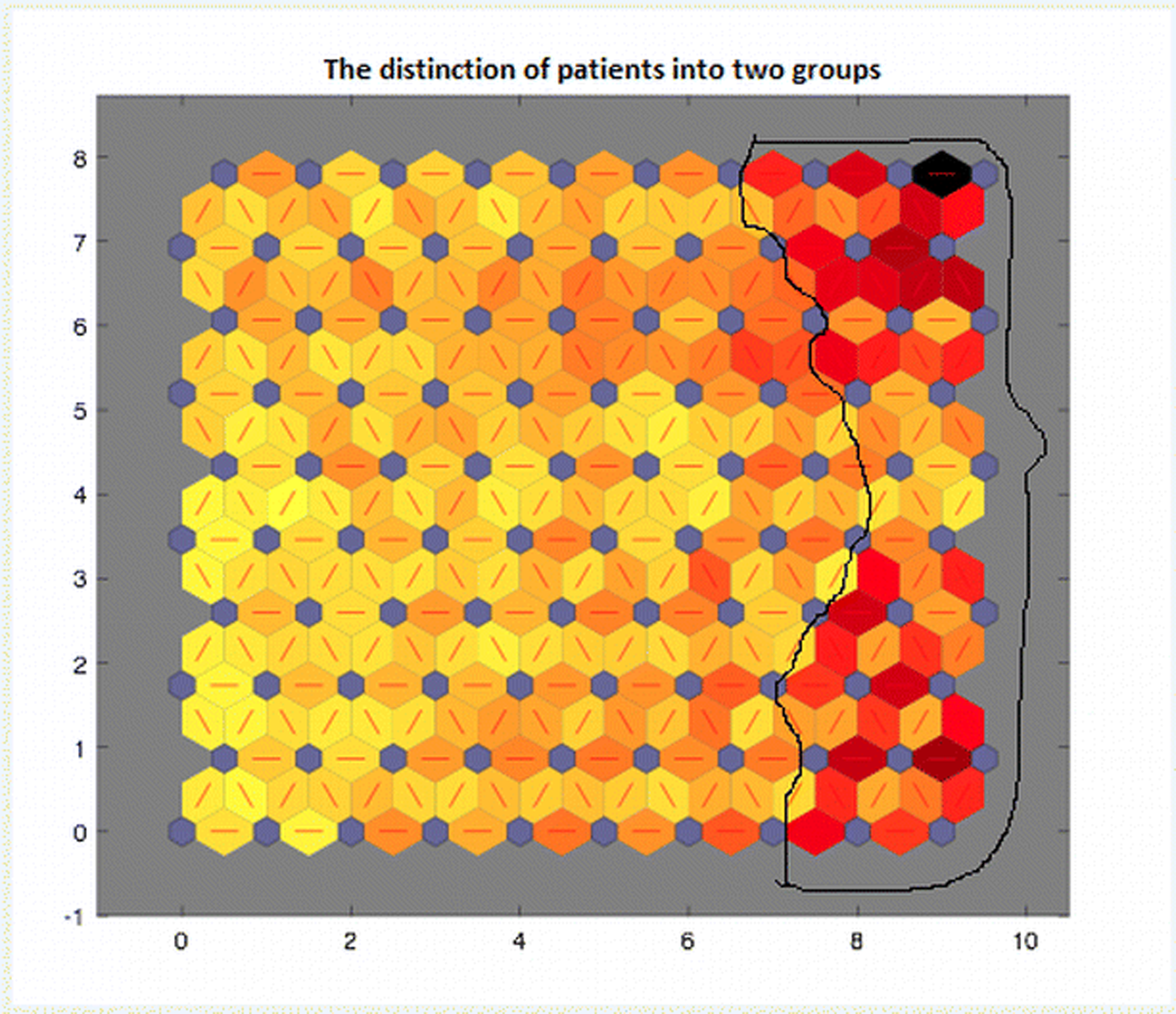

Supplement: Supplementary file 3 — The U-Matrix (weight distance matrix) showing the SOM neighbor weight distances. (GIF 136 kb) [file 428_2019_2642_Fig6_ESM.png]

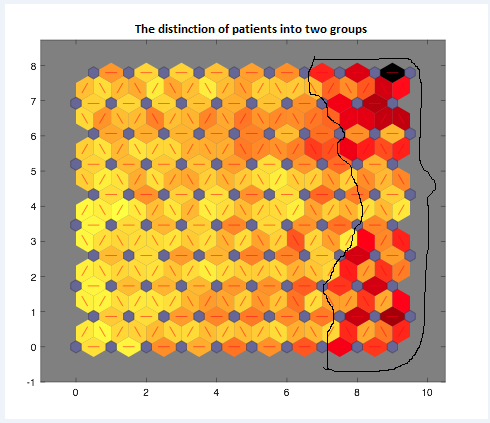

Supplement: Supplementary file 4 — High resolution image (TIF 109 kb) [file 428_2019_2642_MOESM2_ESM.tif]
